# The use of ALS, botanical, and soil data to monitor the environmental hazards and regeneration capacity of areas devastated by highway construction

**DOI:** 10.1007/s11356-015-5637-6

**Published:** 2015-11-03

**Authors:** Dominik Kopeć, Beata Woziwoda, Jacek Forysiak, Łukasz Sławik, Agnieszka Ptak, Edyta Charążka

**Affiliations:** Department of Geobotany and Plant Ecology, Faculty of Biology and Environmental Protection, University of Lodz, Banacha 1/3, 90-237 Lodz, Poland; Department of Geomorphology and Palaeogeography, Faculty of Geographical Sciences, University of Lodz, Narutowicza 88, 90-139 Lodz, Poland; MGGP Aero Ltd., Słowackiego 33-37, 33-100 Tarnów, Poland

**Keywords:** River valley, LiDAR, DTM, Natura 2000, Viaduct, Invasive species, Restoration, Floodplain

## Abstract

The impact of viaduct construction on the vegetation of a river valley was studied in Central Poland (Natura 2000 site PLH100006). The research aimed at assessing the suitability of ALS (airborne laser scanning), soil, and botanical data for monitoring the environmental effects of right-of-way reclamation 1 year after the road construction. Based on the data mentioned above, the following problems were identified: changes in topography and hydrological conditions of the valley as a result of improper land levelling, the use of inadequate soil for reclamation, no spontaneous regeneration of natural vegetation along the entire right-of-way, as well as the abundant occurrence of invasive species. The results of analysis were used to define strategies for mitigation of adverse impacts of the viaduct construction.

## Introduction

Roads are an increasingly common feature of the contemporary landscape, and the number and range of roads have dramatically increased during this century (Laurance et al. 2014). While serving the people as transportation corridors, they negatively affect the natural environment (Spellerberg 1998; Trombulak and Frissell 2000; Seiler 2003; Van der Ree et al. 2015). Roads are a major proximate driver of habitat loss and landscape fragmentation (Li et al. 2005; Tillmann 2005; EEA 2011). As linear structures, they create physical barriers which negatively affect the distribution of animal and plant populations (Reijnen et al. 1995; Angold 1997; Rondinini and Doncaster 2002; Jaeger et al. 2005). However, roads can serve as migration or dispersal corridors for wildlife, including exotic species (Parendes and Jones 2000). Being the links between habitat patches, they may positively contribute to the ecological quality and resistance of the wider environment (Cranmer et al. 2011). Roadsides are excellent habitats or refugia for certain species (Bennett 1991; Merriam 1991; Parendes and Jones 2000; Clark et al. 2001; Gelbard and Belnap 2003; Hansen and Clevenger 2005; Thiele et al. 2009), and proper management of road verges may even support the conservation of rare and threatened plant and animal species (Meunier et al. 1999; Cousins and Lindborg 2008; Milton et al. 2015).

Roads affect and transform physical and chemical conditions of adjacent habitats (Flanagan et al. 1998; Findlay and Bourdages 2000; Trombulak and Frissell 2000). The major changes are observed within a lane road and within a 200-m zone along the road. However, changes may occur many kilometers away from a road, and with consequences that go beyond the time of the road’s construction (Richardson et al. 1975; Forman and Alexander 1998; Forman 2000; Jones et al. 2000). Construction and maintenance of roads transform the wetlands and might cause many ecological effects. The most important are habitat loss, habitat disturbance, mortality of animals, migration barriers, and behavior modification (Li et al. 2014).

Poland, like other Central and Eastern European countries—new EU members, expands its national and international road network. Over 1300 km of motorways and nearly 1400 km of expressways were built in the last decade and the construction of further 1,770 km is planned by 2020 (National Road Construction Programme for 2014–2020 (2014)). Rapid development of road infrastructure poses a threat to nature. The efforts to minimize the negative impacts of roads and connecting transportation services on the natural environment and ecological services are included in the requirements and policies of road construction (Maranda 2011; Nowacka 2014), but the undertaken measures are still insufficient (Solon 2013).

If possible, the construction of roads running through conservation areas is avoided. However, taking into account the fact that the protected areas cover approximately 33 % of the country, it is not possible to completely eliminate the conflict between roads and protected areas (Jędrzejewski 2009; Chmielewski and Kolejko 2014). Similarly, the existing dense network of rivers generates numerous road/river conflicts, while the direct and indirect impact of roads on the valuable river ecosystems is one of the most serious threats (Thrasher 1983; Findlay and Bourdages 2000). In Poland, the intersections of roads with river valleys occur approximately every 4.4 km, and a typical river crossing is 3- to 5-m wide (Maranda 2011). Such conflicts represent 80 % of the total number (4700) of all road/river channel intersections. However, the road construction affects not only the river channel but also the river valley which is usually occupied by valuable habitats and represents the only well-preserved ecological corridor in the cultural landscape (Jones et al. 2000). A number of mitigation measures are undertaken to minimize the negative impacts and to preserve the biotic integrity of the river valley ecosystems and their function as wildlife corridors (Goodrich-Mahoney 2002; Rudnick et al. 2012). The construction of trestle bridges (viaducts) is one of them (Jackson and Griffin 2000).

We studied the impacts of road construction on a lowland river valley, which is part of the Natura 2000 site. The purpose of this paper is to provide the information for the analysis of ecological impacts exerted by activities related to highway development and the evaluation of related ecosystem mitigation measures. The assessment of short-term (1 year after the road construction) ecological effects of the road construction leads us to planning of effective restoration in the near future. We used three sources of data in this analysis: aerial, soil, and botanical. The airborne laser scanning was planned for objective and quick analysis of changes in the topography. The soil research was designed to assess the changes in habitat conditions. The botanical data provided information about the condition and regeneration capacity of the vegetation cover. The acquired data are thus related to transient conditions prevailing under the viaduct in a short period of time after the disturbance.

## Material and methods

### Study area

#### Characteristics of the river valley

The study was carried out in the central part of Poland (Central Europe) along the middle section of the Bzura River (the left-bank tributary of the Vistula). The area is protected through the Natura 2000 network of protected sites under the Habitats Directive (PLH100006) and Birds Directive (PLB100001). The Bzura is a lowland river over its entire course. The analyzed section of the Bzura uses the floor of the Warsaw-Berlin Marginal Valley (Jewtuchowicz 1967). The valley floor was covered with peat in the early Holocene. At present, the thickness of sedge and sedge-reed peat ranges from several tens of centimeters to 3 m, and its top part is highly decomposed. The road viaduct intersects with the Bzura River valley perpendicularly and the valley floor at this point is approximately 1500-m wide and is almost entirely composed of peat. The Bzura River bed runs through a man-made canal. Valley slopes are built of till, and the southern slope is steeper. Flattened moraine areas extend over the river bed in the form of erosional terraces of the marginal valley. For centuries, the valley floor has been exposed to strong human impact. The biggest changes occurred in the Bzura River valley along the stretch Łęczyca–Łowicz where extensive regulatory work was related to strong paludification. Since 1823, the Bzura River bed has been deepened, straightened, and a number of small drainage ditches have been dug (Kobojek 2004). The excessive draining in the valley bottom led to desiccation of soils and wetland habitat degradation (Olaczek 2002; Kopeć et al. 2014a).

At present, the vegetation of the valley floor is homogenous (Kopeć et al. 2014b). The dominant vegetation consists of sedge communities which form a multi-species mosaic of eutrophic wetland communities and wet meadows. As a result of agricultural land-use abandonment, large phytocoenoses of meadows are replaced by willow shrubs *Salix cinerea* and floodplain forests composed of black alder *Alnus glutinosa* in the process of ecological succession. The section of the marginal valley under study is dominated by *Carex acutiformis*, *Carex riparia*, *Phragmites australis*, and *Glyceria maxima*. Phytocoenoses of these communities are dominant on both sides of the existing right-of-way, and they formed homogeneous phytocoenoses before the highway construction.

#### The history of viaduct construction

The study area was an approximately 2-km section of the A1 highway (starting point 263 + 307 km; end point 264 + 980 km), which is part of European route E75 situated within Pan-European transport corridor VI. In this section, the highway intersects with the floor of the Warsaw-Berlin Marginal Valley and is routed on a viaduct. Delimitation of the new highway was preceded by variant analysis under the Environmental Impact Assessment (EIA). The EIA procedure imposed on the investor the necessity to build the highway on the viaduct to significantly reduce the project’s impact on the environment, particularly on the wetland ecosystems developing on the floor of the marginal valley and protected as a Natura 2000 site. The construction works started in July 2010 and were completed in November 2012 (Table 1).

**Table 1:**
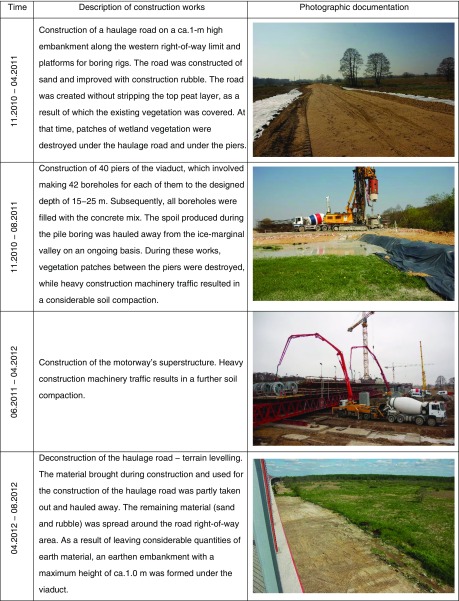

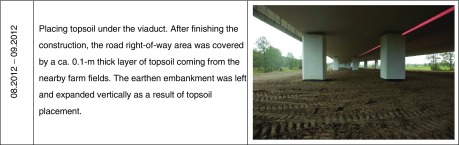
Description of the stages of viaduct construction which contributed to the present conditions in the road right-of-way

### Methods

**Fig. 1:**
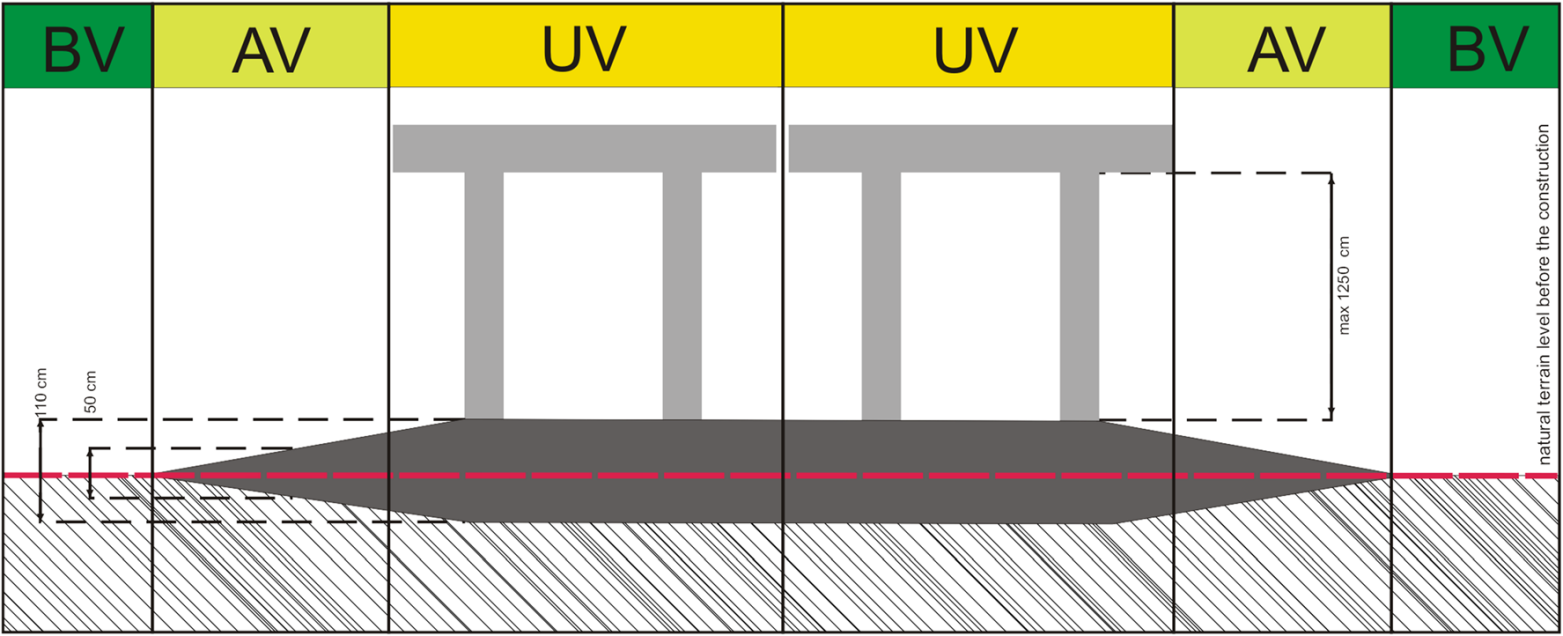
Location of the study transects divided into three zones (*UV* under the viaduct, *AV* adjacent to the viaduct, *BV* beyond the viaduct)—transverse projection

The study area was divided into three zones, different in terms of the disturbance extent and the current habitat conditions. The zones covered the area from the viaduct axis to the areas situated beyond the right-of-way (Fig. 1).

Zone 1—under the viaduct (UV). An approximately 18-m-wide area directly under the viaduct, totally devastated during the construction of the bridge.

Zone 2—adjacent to the viaduct (AV). An approximately 15-m-wide area within the right-of-way but outside the inside diameter of the viaduct, totally devastated during the construction of the bridge.

Zone 3—beyond the viaduct (BV). An area beyond the right-of-way, in the immediate vicinity of the transformed areas, the valley’s floor covered with peat, not disturbed by the construction of the bridge.

#### Airborne survey

Two LiDAR datasets were used in the presented research. The first one comes from ISOK–IT System of the Country’s Protection against extreme hazards. These data were collected during the initial stage of highway construction, on the 20th of April 2011, using a Leica ALS60 scanner with 4 points per square meter. Therefore, we can observe some roadwork cuttings. The second data collection was performed by MGGP Aero on the 23rd of May 2014, using a Riegl LMS-Q680i scanner with 6 points per square meter, after finishing the construction of the highway.

The raw ALS point cloud was spatially calibrated using RiProcess 1.6.4. The data were converted into a geodetic coordinate system using GPS/INS data and scanning strip adjustment. The accuracy of the process was 1 sigma = 0.05 cm. Then, the point cloud was classified using the TerraScan application from the TerraSolid package (version 14). Each point from the point cloud was assigned to the corresponding land-cover class according to the ASPRS standard (http://www.asprs.org) (1—processed, unclassified; 2—ground; 3—low vegetation; 4—medium vegetation; 5—high vegetation; 6—buildings; 7—noise), with separated buildings and ground and vegetation cover. The digital terrain model (DTM) was produced using the TerraSolid software by creating a raster grid of 0.5-m cells based on a triangle model generated from the point cloud classified as class 2—ground.

The point cloud provides the 3D information about the actual situation (DTMs are only 2.5D). Using this source for the creation of a profile, we obtain the complete information about the vertical distribution of points representing the vegetation and the terrain underneath. The location of the profile was selected in the area which in 2011 was still unaffected by the highway construction.

#### Soil survey

To provide the description of the thickness of the embankment layer and the material used to build it, as well as the thickness of the peat layer covered by the embankment, hand-boring was carried out in 2014 using an Edelman auger and an Eijkelkamp gouge auger. The embankment was penetrated by hand-boring using a gouge auger to provide a description of biogenic deposits. Drilling was conducted along seven transects perpendicular to the viaduct axis, adjacent to the botanical transects. Drilling was carried out in the embankment axis—the UV zone, in the UV/AV transition zone, in the middle of the AV zone and beyond the embankment within the BV zone. In addition to the assessment of the embankment thickness and the thickness of biogenic deposits, a description of the deposits from the peat layer was provided. Drilling was continued up to a depth of 3 m. In July 2015, complete sediment cores were collected in four research profiles, two in the UV area and two in BV. In a laboratory, fresh samples of a specific volume were collected—33 from the UV zone and 38 from the BV zone. The bulk density was determined as a dry-matter weight in a fresh sample of a specific peat volume.

#### Botanical survey

**Fig. 2:**
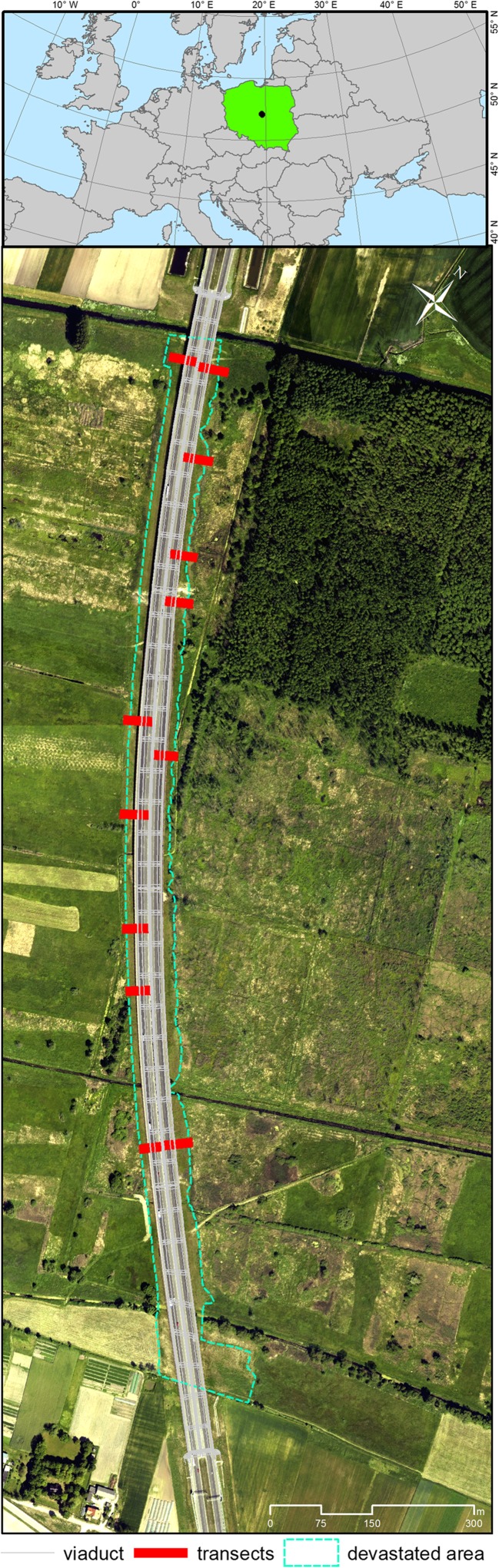
Location of the study area and transects for botanical studies

Botanical studies were conducted in 2013, i.e., 1 year following the completion of the viaduct. The transects were arranged perpendicularly to the viaduct axis. Each of the 12 transects started in the highway axis and ran perpendicularly towards the eastern or western end of the right-of-way (6 transects on each side; Fig. 2). The transects were evenly distributed within the study section of the highway and each of them was situated in the middle section between two pillars of the viaduct. The length of the transects varied and was adjusted to the width of the right-of-way. Each transect covered all three zones (UV, AV, BV) and ended within semi-natural communities beyond the right-of-way. The transects consisted of adjacent 2 × 2-m study plots.

Botanical studies were based on the homogeneity of patches. The cover of all vascular plant species (herbs, shrubs, and trees) was assessed in each 4-m^2^ plot, using a 10° cover-abundance scale—from “1” for species covering 1–10 % to “10” for plant species covering more than 90 % of the plot area—which enabled us to assess both the number of species and the percentage of the area covered by each species.

#### Statistical analysis

Statistical differences in the mean peat compaction in two groups (UV and BV zones) were assessed with the independent *t* test. Before this analysis, the normality was checked with the Shapiro-Wilk test and the homogeneity of variance with Levene’s test.

The ecological profile of floras and habitats was analyzed using the ecological indicator values (EIV) for light and moisture requirements taken from Ellenberg et al. (1992). The species defined in the Ellenberg’s system as indifferent species and non-classified (undetermined) were excluded from the analysis. Mean indicator values were calculated for each sampling plot on the basis of all species present in a sample, taking into account cover values as weights. For each plot, the following was also calculated: the number of apophytes (native disturbance-adapted plant species), species invasive in Poland (Tokarska-Guzik et al. 2012), and target species. Target species were those indigenous reed-bed species that occur naturally in semi-natural communities of the marginal valley (Kopeć et al. 2014b).

The data were then used to calculate an average value of Ellenberg’s indicator values and the average number of apophytes, invasive species, and target species for all plots within the 12 study transects located at the same distance from the highway axis.

Statistical differences in the number of apophytes and IAS (invasive alien species) and Ellenberg’s indices were assessed with the Kruskal-Wallis test and the Dunn post hoc test (for >2 independent groups of data). The significance level was set at 0.05. As the data did not exhibit a normal distribution, non-parametric tests for this analysis were used. These statistic comparisons were carried out using the SPSS software package (version 23.0, SPSS Inc., USA).

Indicator values (IndVals) (Dufrêne & Legendre, 1997), calculated using the PC-ORD 6.15 software (McCune & Mefford, 2011), were used to identify plant species significantly associated with each zone (UV, AV, BV).

## Results

### Description of changes in the land relief

#### ALS results

**Fig. 3:**
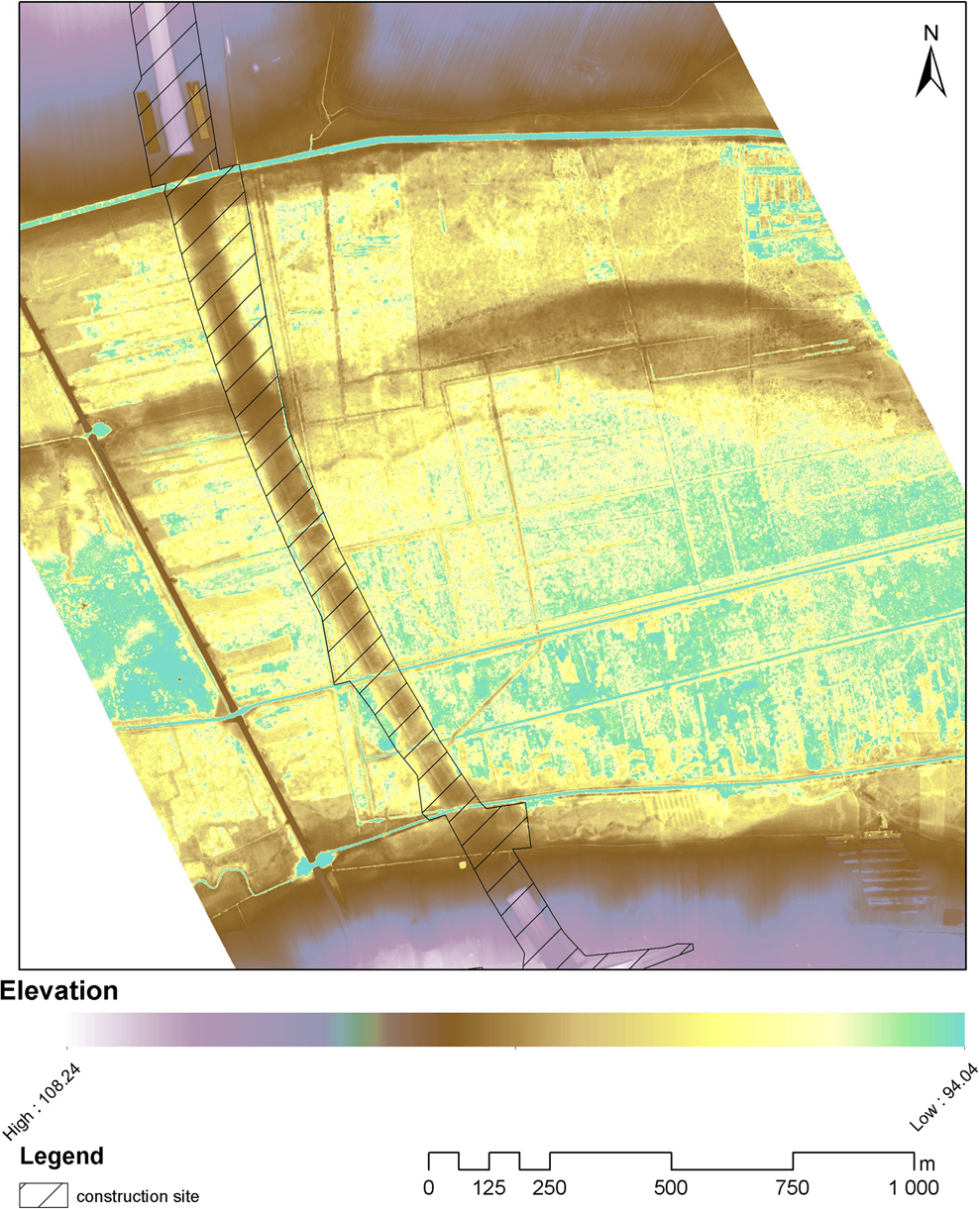
Digital terrain model created from the point cloud collected in 2014—after the highway construction)

**Fig. 4:**
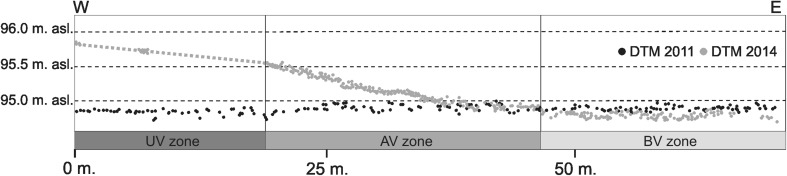
Profile for two point clouds from 2011 and 2014

DTM produced in 2014 shows an embankment along the entire right-of-way that runs across the river valley floor (Fig. 3). The embankment is currently elevated by a maximum of approximately 1 m over the natural terrain elevation (max. 95.8 m above sea level) and is approximately 80-m wide. The embankment is situated within the right-of-way. The terrain elevation profile across the right-of-way created in 2011 and 2014 shows that the embankment was built during the highway construction and is located in what used to be a flat area at approximately 94.8 m above sea level before the project has started. The embankment reaches the maximum height within the highway’s axis and then gradually descends to approximately 0.5 m at the borderline of the UV/AV zones and then further descends up to the end of the right-of-way (AV/BV zones’ borderline) to disappear completely (Fig. 4).

#### Results of soil studies

**Fig. 5:**
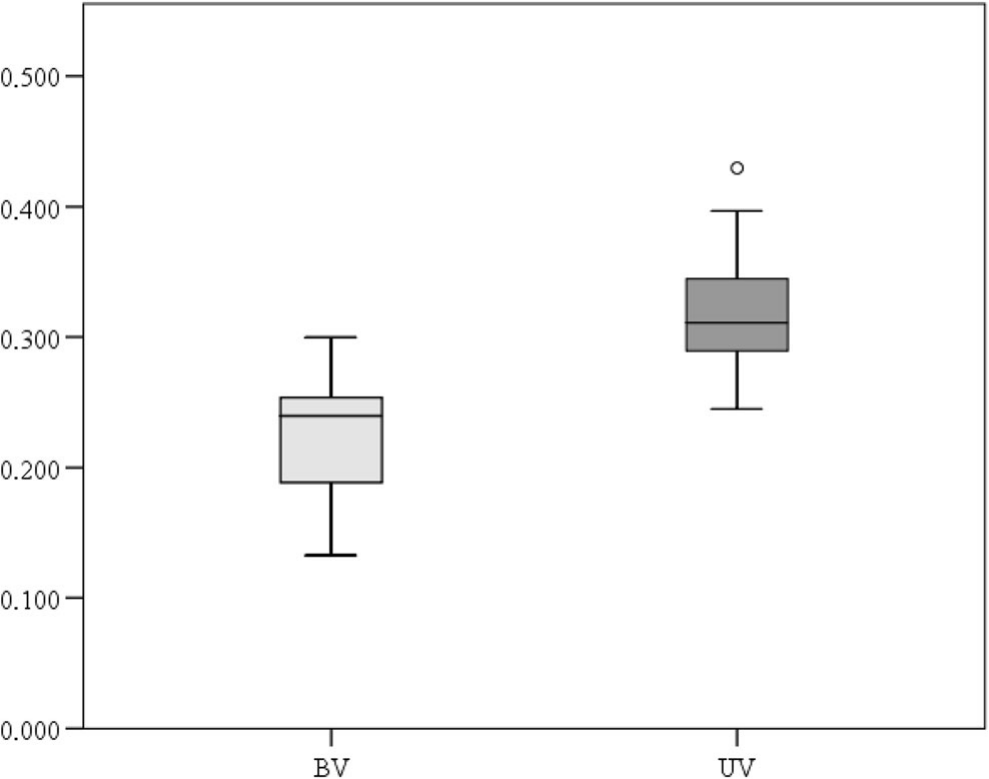
Comparison of the peat bulk density between the UV and BV zones. The average bulk density was significantly different, according to the independent *t* test (*t* = 9.431, *p* < 0.005)

As evidenced by drilling carried out in the UV zone, the maximum thickness and the maximum height of the embankment is located under the viaduct. The embankment is composed of sand and concrete rubble which in many places form a solid cover. The embankment’s thickness in the viaduct axis ranges from 60 to 140 cm, on average ca. 100 cm. Further from the viaduct axis, the thickness of the soil overlay decreases, ranging from 55 to 100 cm and reaching approximately 70 cm at the borderline of the UV/AV zones. A highly decomposed peat layer (black color) is deposited directly under the embankment, underlain by brown herbaceous peat whose substratum is composed of organic mud or mineral sand with mud. The total thickness of biogenic deposits varies at different sites along the viaduct and ranges from 40 to 175 cm. The average thickness of peat and organic mud in this zone is 80.5 cm. The peat under the embankment is highly compressed. The mean bulk density of peat in the UV zone is 0.318 ± 0.041, while in the BV zone—0.223 ± 0.043 (Fig. 5). The difference between these values is statistically significant (*t* = 9.43; *p* < 0.001).

In the AV zone, the thickness of the embankment is much smaller and clearly decreases towards the end of the right-of-way. The overlay is composed mainly of sand, with insignificant contribution of rubble. The overall thickness of the overlay in the middle of the AV zone ranges from 20 to 60 cm, on average 40 cm. Biogenic deposits under the embankment consist of peat and the underlying organic mud, the overall thickness of which is on average 77.5 cm. The embankment ends at the borderline of the AV/BV zones.

The BV zone includes the peat-covered valley floor outside the embankment. This zone is dominated by highly decomposed peat (black color) and slightly decomposed peat (brown color). In some profiles, highly decomposed peat is deposited directly on the surface, in others—it is covered with slightly decomposed herbaceous peat. In some profiles, organic mud was found under the peat. The total thickness of biogenic deposits in this zone ranges from 85 to 160 cm, and on average 103 cm, which means 20 cm more than under the embankment.

#### Plant species composition

**Fig. 6:**
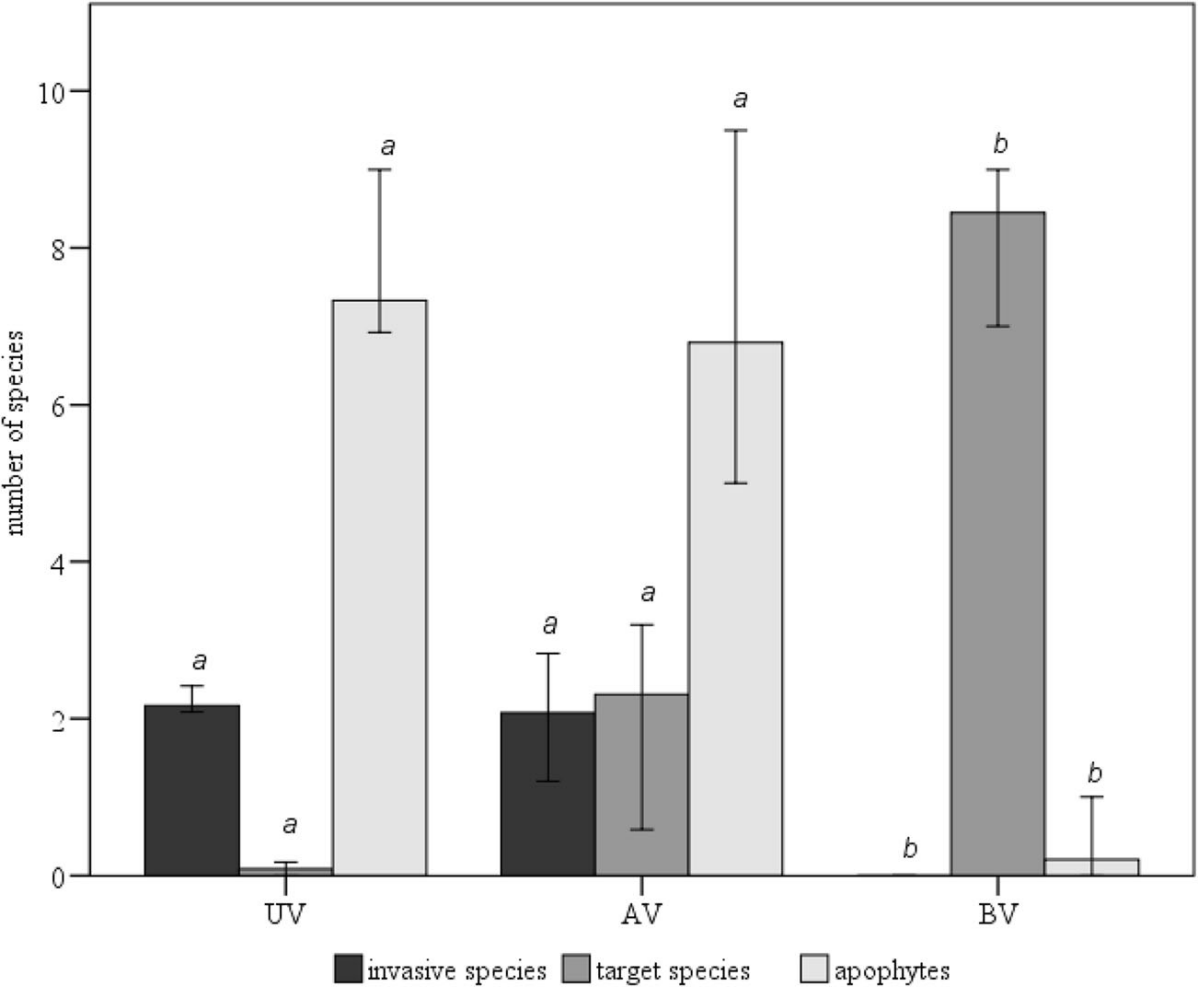
Comparison of the number of apophytes (*H* = 10.186, *p* < 0.05), invasive (*H* = 9.981, *p* < 0.05), and target species (*H* = 20.912, *p* < 0.001) between the UV, AV, and BV zones. *Boxes with the same letter* are not significantly different, according to the Kruskal-Wallis and Dunn post hoc tests

**Fig. 7:**
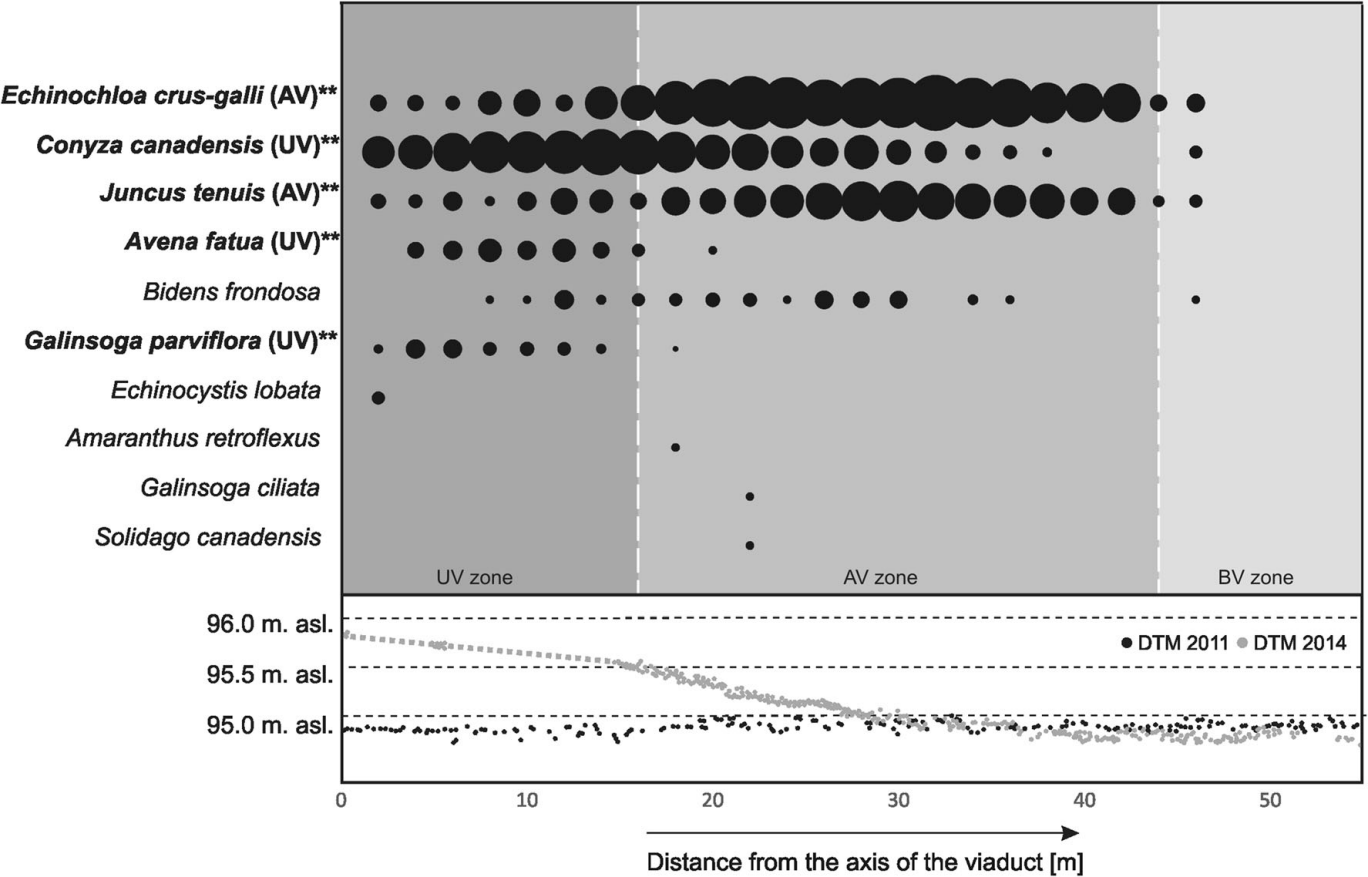
Average percentage cover of individual invasive species at sites located at certain distances from the highway axis. Values calculated for 12 transects. The *area of a circle* corresponds to the average percentage cover of a given species at a given site. *Species in bold* are significantly correlated (IndVal analysis) with one of the three zones (*p < 0.05, **p < 0.005); a code of this zone is provided in *brackets*. In the *lower part of the figure*, cross-section elevation profiles are presented, prepared by laser scanning using the data collected in 2011 and 2014

A total of 170 species of vascular plants were identified in 2013 in 254 patches located in 12 transects. Most of them were species characteristic of anthropogenic habitats, including a large group of apophytes and invasive species (IAS) (Figs. 6 and 7).

The analysis of the distribution of apophytes and IAS in the profile running across the right-of-way showed that their highest average number occurred between the 14th and 30th meter from the viaduct axis at the borderline of the UV/AV zones. The average number of apophytes and IAS not different significantly between UV and AV zone (Fig. 6). The highest density of these species was observed (apophytes—10 species/4 m^2^ and invasive species—3.1 species/4 m^2^) in areas where the embankment is approximately 0.4-m high and the light penetration is only slightly limited by the viaduct. Invasive species and apophytes occur only at sites located in the immediate proximity of the right-of-way, with small density in the BV zone. The average number of apophytes and invasive species in BV zone was significantly lower then in UV and AV zone (Fig. 6).

Among the invasive species, *Galinsoga parviflora*, *Avena fatua*, and *Conyza canadensis* were significantly correlated with the UV zone (Fig. 7). The maximum average cover of these species was between the 4th and 10th meter from the viaduct axis (2.2, 2.5, and 12.5 %, respectively). The AV zone was invaded by *Echinochloa crus-galli*, which occurred with the highest percentage cover between the 22nd and 34th meter from the viaduct axis (where the embankment height was below 0.4 m). Its cover ranged from 14.5 to 21.3 %. *Juncus tenuis* was yet another species noted with the maximum cover in the AV zone. Both of them are statistically significantly associated with the AV zone (Fig. 7). The average cover of this species reached the maximum values ranging from 7.0 to 9.5 % between the 26th and 34th meter from the viaduct axis.

Four invasive species were identified in the BV zone: *E. crus-galli*, *C. canadensis*, *J. tenuis*, and *Bidens frondosa*. They occur with the maximum percentage cover of 2.0 %, no farther than 4 m from the borderline of the AV/BV zones. The frequency and density of *B. frondosa* were similar in all three zones, and its maximum cover did not exceed 1.6 %.

The analysis of the target species distribution indicated that most of them occurred only in the BV and AV zones (Fig. 6). The average number of target species in the UV and AV zones was not significantly different (Fig. 6). *Lythrum salicaria*, *Rorippa palustris*, and *Polygonum amphibium* (Fig. 9) were also found in the UV zone, but their average cover was below 2.0 %. Rush species were much more frequent and abundant in the AV zone. Two of them—*L. salicaria* and *Juncus articulatus*—were identified as indicators of this zone. Generally, a significantly larger contribution of target species was observed in plots situated farther than 24 m from the highway axis where the embankment was not higher than 0.2 m. *L. salicaria*, *Typha latifolia*, *R. palustris*, *Alisma plantago-aquatica*, and *J. articulatus* were frequently observed in the zone located between the 24th and 44th meter from the highway axis. Among these species, *L. salicaria* reached the highest average cover (33.0 %) at the sites situated 40 m from the viaduct axis, and *T. latifolia* (with average cover of 21.3 %) at the sites situated 42 m from the viaduct axis.

**Fig. 8:**
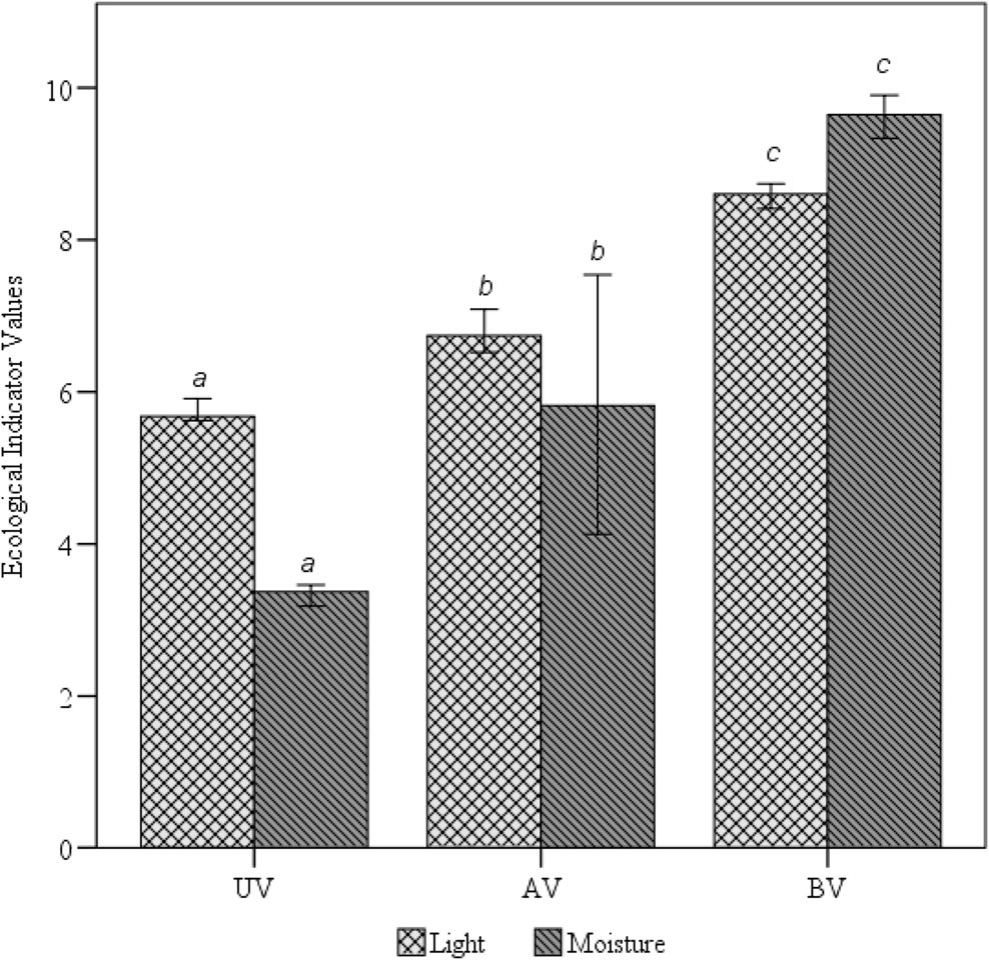
Comparison of the light (*H* = 21.257, *p* < 0.005) and moisture (*H* = 21.358, *p* < 0.005) EIVs between the UV, AV, and BV zones. *Boxes with the same letter* are not significantly different, according to the Kruskal-Wallis and Dunn post hoc tests

Light and moisture EIVs varying in the cross-section profile had a similar pattern. The average EIVs of light and moisture were significantly different in all three zones (Fig. 8). Extremely low values were recorded in the UV zone, where moisture values ranged from 3.1 to 3.8, and were associated with the embankment of over 0.5 m in height. The value indicated that the conditions were favorable to dry-site species. Light indicator values under the viaduct ranged from 5.6 and 6.0, which corresponds to the conditions favorable to semi-shade plants. The AV zone had higher increases in the moisture and light EIVs; however, the average values of these indicators did not reach the values characteristic of the BV zone. In the BV (reference) zone, the moisture indicator values ranged from 9.0 to 10.0, and light indicator values ranged from 8.3 to 8.8, which corresponds to wetland conditions and intense sunlight.

## Discussion

### Soil and ALS data

Soil and remote sensing (ALS) studies demonstrated that two barriers were created across the river valley as a result of the viaduct construction. The barriers were not removed during the land reclamation, and 1 year after the project completion, they still pose a serious threat to the valley ecosystems. The first barrier is the embankment (Figs. 3 and 4), and the other one is compressed peat under the bridge.

Peat and organic mud that build the valley floor in the viaduct zone are deposits of high porosity and water capacity (Charman 2002). Therefore, natural conditions prevailing in the valleys of the Bzura and other rivers of similar origin allow a gravitational, horizontal water flow in the ground (Warburton et al. 2004; Harvey et al. 2009). Peat is compacted as a result of pressure from the overlying deposits (particularly the mineral ones) (Hartlén and Wolski 1996; Wrzesiński and Lechowicz 2012). Consequently, it is characterized by higher density and smaller porosity, and a decreasing volume (Skempton 1970; Baldwin 1971). Studies conducted in the Bzura valley showed that covering the peat with the material forming the embankment within the UV and AV zones reduced the thickness of the peat layer and decreased the altitude of its original surface by 0.2–0.4 m. This is due to the susceptibility of peat to the compaction. Sedge peat and reed peat occurring in the study transect are characterized by a high permeability coefficient (Eggelsmann 1980; Rydelek 2011). Acrotelm (the upper active layer of peatlands) has the highest permeability, which decreases with natural autocompaction (Hoag and Price 1995; Charman 2002). In the case of compacted peat, as a result of geotechnical work, the permeability coefficient increases several times (Wielechowska and Mioduszewski 2003). The pressure caused an increase in the bulk density of peat. The comparison of peat samples from the area under the viaduct (UV) and samples from the zone beyond the viaduct (BV) indicated an increase in the bulk density of about 142 %. Consequently, the horizontal water flow in the ground is reduced. The embankment across the valley floor has a similar effect as it hinders free migration of surface water, which greatly affects the natural vegetation of the river valley floor (Bunn and Arthington 2002; Tockner and Stanford 2002). It may affect the local hydrology by reducing the water permeability of the soil, affecting the natural moisture balance and reducing the water inflow (Price and Schlotzhauer 1999; Charman 2002; Rydelek 2011). In the case of wetlands fed by groundwater seeping in the basin slope zone, the existence of a barrier in the form of an embankment has a limited effect on the groundwater level (Wielechowska and Mioduszewski 2003). However, it is very important to the water filtered through the peat deposit or running off the surface, which is the case at the study site. The water level will probably rise in front of the embankment and lower below the embankment (Hartlén and Wolski 1996). The permanent lowering of the water table in some places and the rising water table in others will basically affect the composition of plant communities in the long term. However, the area and the extent of changes, and the response of the ecosystem to the construction of the road will greatly depend on the relief of the valley floor (Fig. 3). For example, the presence of the artificial canal which runs across the UV zone will mitigate the negative impacts of the physical barrier.

### Botanical data

**Fig. 9:**
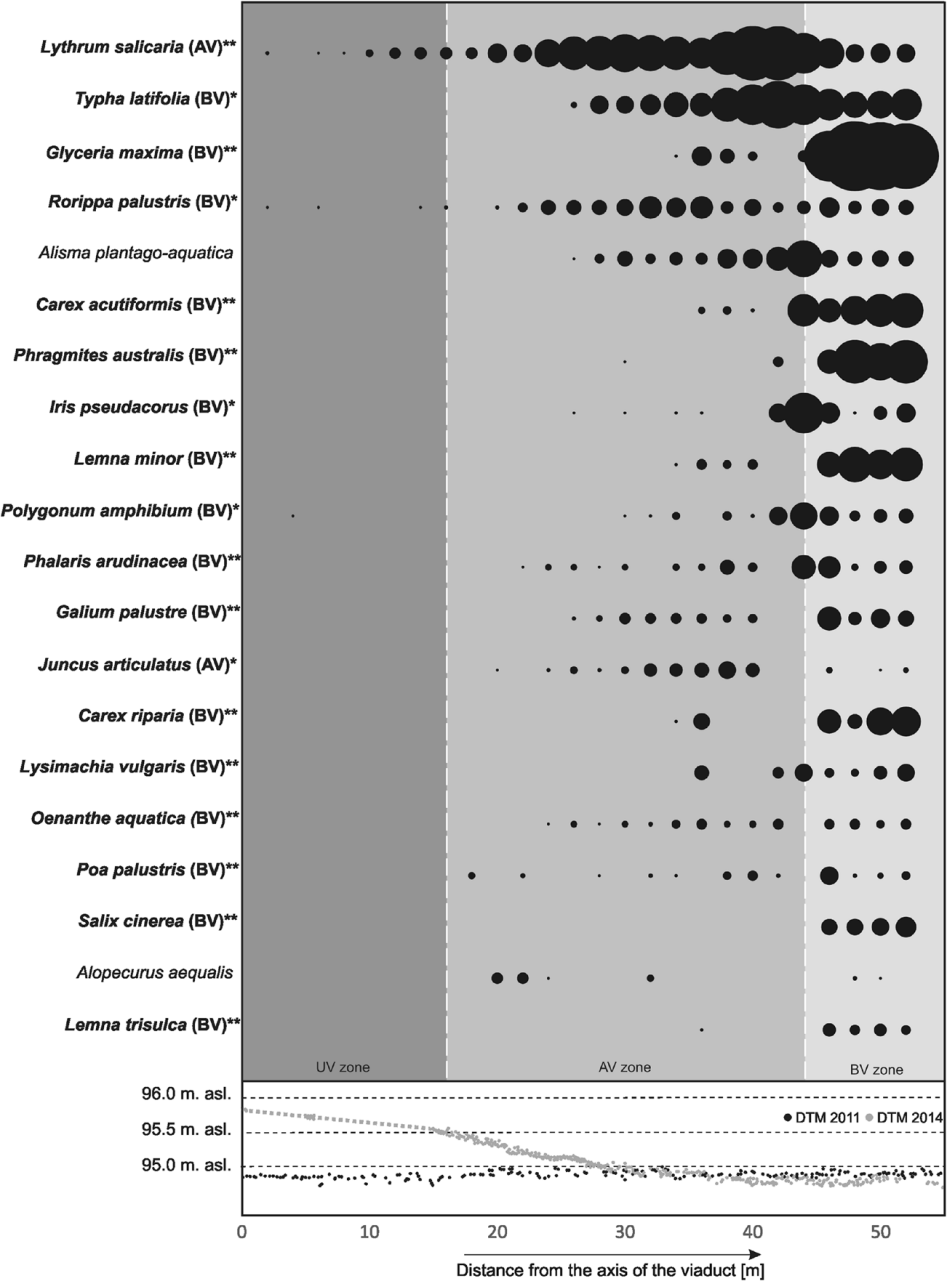
Average percentage cover for individual target species at sites located at certain distances from the highway axis. Values calculated for 12 transects. The *area of a circle* corresponds to the average percentage cover of a given species at a given site. *Species in bold* are significantly correlated (IndVal analysis) with one of the three zones (*p < 0.05, **p < 0.005); a code of this zone is provided in *brackets*. In the *lower part of the figure*, cross-section elevation profiles are presented, prepared by laser scanning using the data collected in 2011 and 2014

The results of the botanical research indicate that 1 year after the road construction, the plant species composition of the road zones (UV and AV) is significantly different from the species composition of the peat-covered river valley zone (BV). Large differences in the floras of the UV/AV zones and the BV zone indicate that there are factors reducing the regeneration of rush vegetation (Figs. 6 and 9) in the degraded area. A short time of the natural vegetation regeneration is obviously one of them. The analysis of the relationship between the height of the embankment and the penetration of the target species shows a clear decrease in the number of target species in the section of the embankment higher than 0.2–0.3 m. This is mainly connected with the low moisture content in the substrate of the highest sections of the embankment (Fig. 8). Based on the analysis of Ellenberg’s ecological indicator values, it has been demonstrated that the conditions are favorable to species which prefer mesic (not wet) habitats. The degraded area is dominated by disturbance-adapted plant species (Figs. 6 and 7). Some of them (*C. canadensis*, *A. fatua*, *G. parviflora*) are invasive alien species in Poland (Tokarska-Guzik et al. 2012). Their propagules might be transported from surrounding arable fields with the soil used in reclamation (Table 1). However, it is also possible that the identified alien plant species used this linear structures—the man-made road and the natural river valley—as migration corridors (Pyšek and Prach 1994; Collingham et al. 2000; Hansen and Clevenger 2005; Dajdok and Pawlaczyk 2009), open for 2 years during the viaduct construction. This “new land” patch established during the road building, isolated from vegetation, was easily colonized by invasive and disturbance-adapted plants (Figs. 6 and 7). The presence of invasive species (*B. frondosa*, *J. tenuis*, and *E. crus-gali*) in the AV zone, between 0.0 and 0.3 m of the embankment’s height, poses a particularly serious threat because rush vegetation in this section regenerates with target species. The species are highly expansive in the Bzura valley (Kopeć et al. 2014a, Kopeć et al. 2014b). Due to the competition, they may reduce the development of target species and disturb the restoration process (D’Antonio and Meyerson 2002). This may lead to the development of new plant communities different from those suitable for the lowland river valleys (Richardson and Gaertner 2013; Tischew et al. 2014). This direction of the wetland vegetation development in heavily altered ecosystems (in the embankment’s section between 0.0 and 0.3 m) is further confirmed by varying contribution of individual target species in relation to reference sites. The most expansive target species include purple loosestrife *L. salicaria* and *T. latifolia*, which currently dominate in the UV and AV zones. The dense cover of the above species in this zone is related to their life strategy. *Typha* ssp. can spread quickly and extensively, both through aggressive rhizomatous growth and through sexual reproduction (Smith 1967; Shih and Finkelstein 2008). Purple loosestrife is also an expansive species which can spread quickly. It is an invasive and non-indigenous plant species in North America (Blossey et al. 2001; Bastlová and Květ 2002).

The conducted analysis of the flora shows that weed species occur in the BV zone (Figs. 6 and 7). *B. frondosa* and *J. tenuis* observed in the BV zone already 1 year after the construction was completed, and which migrated from the UV and AV zones, are particularly dangerous for the semi-natural wetland communities surrounding the construction site (Fig. 7). The species occur now in wetland communities of the Bzura valley (Kopeć et al. 2014b) and are considered one of the most widespread alien plant species in Europe (Lambdon et al. 2008). They are likely to extend their cover in the BV zone in the following years, displacing the native flora species due to their expansiveness (Otto et al. 1994; Danuso et al. 2012; Myśliwy 2014). The observed changes in BV indicate that the ecological impact of the road with the under-viaduct-embankment involves a considerably larger area than that covered by the road itself (see Wemple et al. 1996; Jones et al. 2000).

The ecological profile of floras in UV, AV, and BV zones is significantly different in terms of light EIVs (Fig. 8). The UV zone with the elevated ground surface is colonized by semi-shade plants (Fig. 8). The further away from the bridge, the better the light conditions and the greater the contribution of light-demanding wetland species. Light conditions prevailing in the UV zone will thus impede the growth of some of the species naturally occurring in the open ecosystem of the BV zone. As light is one of the key factors enabling the growth of species in wetland ecosystems (Kotowski et al. 2001; Kotowski and van Diggelen 2004), species requiring full light conditions will not be able to grow under the bridge, even with the optimum moisture content.

## Conclusion

The highway construction, even over a long viaduct, affected the habitat conditions and the vegetation of the studied section of the Bzura valley, and changes were observed 1 year after the road construction. The obtained results have a preliminary character (only 1 year of observations) and a follow-up of such studies is necessary. Environmentally valuable areas must be monitored to determine the actual environmental impact of the investment upon its completion, based on soil, botanical, and remote sensing studies. This type of studies help to plan corrective actions aimed at rehabilitation of the heavily transformed areas. In the case of the viaduct discussed in this paper, negative impacts are mainly associated with the lack of ecological restoration after the construction process and the lack of detailed construction guidelines implementing relevant standards during the construction. The main threat to the floodplain ecosystem was the failure to restore the land to its natural altitude corresponding to the altitude of the surrounding lands. The embankment served as a temporary road during the construction works, and its reclamation following the road construction should be aimed at restoration of its original altitude and water permeability parameters.

To avoid a similar situation, it is advisable that the soil previously removed from the land covered by construction works is used in the restoration process. It could be collected and stored nearby and returned after the construction. At the same time, the use of soil coming from adjacent farming fields should be forbidden as the soil contains a weed species seed bank.

The results of soil, botanical, and remote sensing studies show that in order to reduce the adverse environmental impact of the investment, it is necessary to implement a number of restoration actions because those applied during the construction works proved ineffective. (a) The most important task is to level the terrain once again. Moisture conditions (Fig. 8) currently prevailing in the UV and AV zones prevent the development of rush vegetation (Figs. 6 and 9). The analysis of the target species distribution in the cross section shows that if the embankment is lowered to the maximum height of 0.2 m, rush vegetation should regenerate spontaneously. (b) The light conditions (Fig. 8) currently prevailing in the UV zone prevent the growth of rush vegetation (Figs. 8 and 9). For this reason, the restoration process should be supported by hay transfer, which is a commonly used method in the restoration of wetlands (Klimkowska et al. 2010; Rasran et al. 2007; Hedberg et al. 2014; Schmiede et al. 2013). The biomass should come from the floodplain forest, and not from open ecosystems adjacent to the construction site (not from the BV zone). The light conditions under the viaduct (Fig. 8) are similar to those prevailing in the undergrowth of floodplain forest (Czerepko 2007), i.e., a climax community of the Bzura valley (Kopeć et al. 2014a). (d) It is also necessary to eliminate weed species from the AV and BV zones. Although their cover-abundance is still small 1 year after the construction (Fig. 6).

The main conclusion is that there was a negative effect of road building on the river valley fens, even though the mitigation measures were implemented. However, the results of ALS, soil, and botanical studies show that this negative effect would be significantly reduced if the embankment at the UV zone is lowered by 0.2 m. It would allow to restore the rush vegetation also under the viaduct, especially if the hay transfer method is implemented. This would allow the UV and AV zones to be overgrown with vegetation whose species composition may be different from the original vegetation in the area and from the vegetation occurring in the BV zone; however, this species composition will mitigate the negative impact of the highway construction.
